# Asbestos-Based Pottery from Corsica: The First Fiber-Reinforced Ceramic Matrix Composite

**DOI:** 10.3390/ma13163597

**Published:** 2020-08-14

**Authors:** Philippe Colomban, Aleksandar Kremenović

**Affiliations:** 1 MONARIS (From the Molecule to the Nano-object: Reactivity, Interaction & Spectroscopies), Sorbonne Université, CNRS, 4 Place Jussieu, 75005 Paris, France; 2 Faculty of Mining and Geology, University of Belgrade, Đušina 7, 11000 Belgrade, Serbia; aleksandar.kremenovic@rgf.bg.ac.rs

**Keywords:** asbestos, CMC, Corsica, fiber, pottery

## Abstract

Asbestos-containing pottery shards collected in the northeast of Corsica (Cap Corse) and dating from the 19th century, or earlier, have been analyzed by SEM-EDS, XRPD, FTIR and Raman microspectroscopy. Blue (crocidolite) and white (chrysotile) asbestos fiber bundles are observed in cross-sections. Most of the asbestos is partly or totally dehydroxylated, and some transformation to forsterite is observed to occur, indicative of a firing above 800 °C. Examination of freshly fractured pieces shows a nonbrittle fracture with fiber pull-out, consistent with a composite material behavior, which makes these ceramics the oldest fiber-reinforced ceramic matrix composite. Residues indicate the use of this pottery as a crucible for gold extraction using cyanide.

## 1. Introduction

The fracture of a ceramic gives rise to a brittle edge, and fracture initiation depends upon the defects and their distribution in the matrix. It is thus difficult to design the shape and mechanical strength of a ceramic object. However, the association of two brittle materials, i.e., a composite material, can give rise to a nonbrittle material which can resist fracture [1,2,3]. Defects no longer determine the ultimate strength of such a material, and it becomes possible to predict the strength that these materials will be able to support [1]. The best fracture resistance is obtained by reinforcing a ceramic matrix with long ceramic fibers, first with boron and carbon fibers, then with fibers having a more complex composition and better thermochemical stability such as glass (silicate E-glass), SiC, Al_2_O_3_ or mullite (3Al_2_O_3_·2SiO_2_) [4]. Natural asbestos fibers have additionally been largely used during the 20th century for the reinforcement of cements—and for thermal insulation and fire-protection—until their toxicity was considered too high in the 1980s. Most scholars believe that fiber-reinforced ceramic matrix composites (CMCs) are an innovation of the 20th century, whereas asbestos-fiber-reinforced pottery was actually produced centuries before in the north part of Corsica (Cap Corse).

The deliberate use of asbestos or similar minerals (serpentines) has been noted since the Middle Ages, but some Neolithic pottery already contained asbestos. Chiva and Ojalvo [5] reported that Alexandre Brongniart, the Head of the famous Sèvres Imperial Factory, known before and then as the Royal Factory, from 1800 to 1847 and founder of the French National Ceramic Museum, first studied asbestos-based pottery from Corsica in an unpublished manuscript [6]. This demonstrates the interest of the founder of ceramic processing science in special ceramics. More recently, ethnological studies on this asbestos-based pottery were published in French in specialized journals [6,7] and drew attention from the ceramic composite community [8]. Archaeological excavations of different sites in Corsica have shown that these asbestos-reinforced artefacts have been produced at least since the Middle Ages in the northeast part of the island, where there are numerous ophiolite and serpentine outcrops (Castagnicia, Nebbio, Monte San Petrone and Cap areas (Canari)), which have been largely exploited during the 20th century for applications in the building and insulation industries. The production of this asbestos-based pottery continued up to the middle of the 20th century. Istria [7] identified at least 15 familial workshops active around 1900. Documents indicate that 3 volumes of “clay” were mixed with 1 volume of “dried asbestos tow”.

Artefacts were used as food cooking utensils. The waterproofing of the porous body (the pottery is not glazed) was accomplished by using sheep fat to plug the pores. Indeed, both the porosity and the fiber reinforcement improve the mechanical toughness of the artefact. According to ethnographic records, the production was conducted mainly by women potters in villages, and the firing took place in bread ovens or in simple pits [7]. Historical records indicate that asbestos-rich pottery was used by the Romans for cremation and that asbestos textiles were made. Hulthén [9] reported that artefacts very rich in asbestos (50% to 90% volume) have been made in Finland and North Scandinavia from ca. 3900 BC to 200 AD and 1500 AD, respectively, for metal production (crucibles and molds). It is also reported that asbestos fibers were added to plaster during the Middle Ages or before in Cyprus [10].

We present here the first study of four shards provided by the Corsican Archaeological Office (DRAC).

## 2. Materials and Methods

### 2.1. Pottery Shards

**Figure 1:**
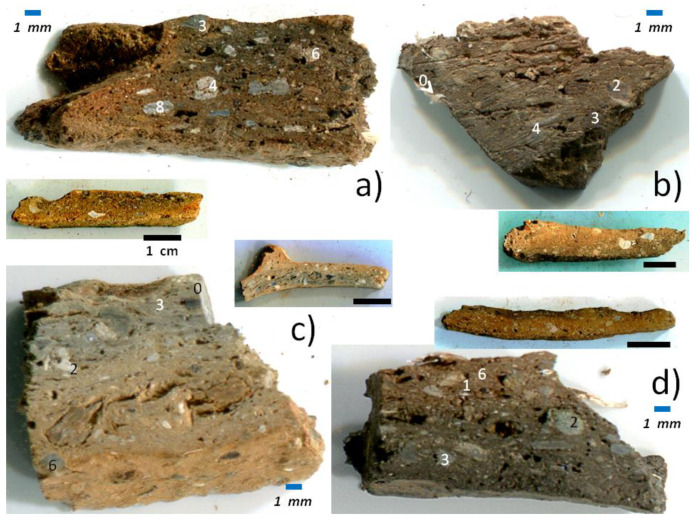
Cross sections of the 4 shards analyzed (**a**–**d**, see Table 1). Raman analyzed spots are labeled.

The four shards have been selected for the variability of their color from a series of samples collected by Corsican scholars in the northeast of Corsica during the 19th and 20th centuries (Table 1). The exact provenances are not known. Figure 1 shows the samples (and cross-sections) and the main spots analyzed by SEM-EDS, XRPD, FTIR and Raman microscopy. Coarse grains (up to 5 mm) are mixed in a rather homogeneous matrix of a high open porosity (~30%).

### 2.2. Methods

The shards were simply cut with a low-speed diamond-rich bronze saw (Minitom, Struers, Denmark) to obtain a flat cross-section. The surfaces and cross-sections were observed with an optical microscope (BX51 Microscope, Olympus Corporation, Tokyo, Japan). Raman microspectroscopic analysis was performed using two instruments: (i) a high-resolution HR800 LabRam spectrometer (Horiba Scientific Jobin Yvon, Longjumeau, France) coupled to a BX Olympus microscope with different long and short working distance objectives, equipped with an Ar^+^ ion laser (457 nm) and a Peltier cooled CCD detector (resolution <2 cm^−1^), and (ii) a high-sensitivity LabRam Infinity (Dilor, Horiba Jobin Yvon, Lille, France) spectrometer equipped with YAG 532 nm and He 633 nm lasers and a similarly cooled CCD detector (resolution ~4 cm^−1^). The analysis was carried out with Olympus ×10, ×50 and ×100 ultralong working distance objectives in order to obtain a characterization of the main phases. More than 20 spectra were collected for each sample with the two instruments. The laser power of illumination at the sample ranged between 1 and 2 mW. Typical counting times ranged between 1 and 30 min. Five to thirty accumulations were made in order to eliminate cosmic events and to increase the signal-to-noise ratios. The Raman study assisted in the identification of representative areas for additional local analyses.

FTIR ATR spectra were recorded on powdered samples with an Alpha (Bruker Optics, Ettlingen, Germany) instrument equipped with Diamond ATR accessory; 30 accumulations were made for each spectrum.

The elemental composition was obtained with a 5410 LV SEM-EDX (JEOL, Tokyo, Japan) using an acceleration voltage of 20 kV. The sample was wrapped with carbon-rich tape with a small window for the area to be studied. Quantitative elemental analysis (oxide) was conducted done using the ZAF calculation method as implemented in the Iridium Ultra software (IXRF Systems, Austin, TX, USA). The validity of measurements was monitored by applying the same procedure to “Corning Museum B, C and D” and American “National Bureau of Standards (NBS 620)” certified glass-reference samples, as usual [11]: the error was estimated to be below 10%, except for aluminum.

X-ray powder diffraction (XRPD) patterns were recorded on powders prepared in an agate mortar with a SmartLab diffractometer manufactured by Rigaku (Rigaku Corp., Tokyo, Japan). CuK_α_ radiation was used. The diffractometer was equipped with a Kβ filter and a D/teX Ultra 250 detector. Data were collected between 5 and 70° 2θ at every 0.01° in the Bragg Brentano geometry. The scan speed was 5°/min and the slit configuration was as follows: incident slit, 0.5°; length limiting slit, 10 mm; receiving slit #1, 20 mm; and receiving slit #2, 20 mm. All specimens were rotated during data collection with a speed of 60 rpm. PDXL 2 software (version 2.8.3.0), Rigaku Corp., Tokyo, Japan) was used for the powder diffraction data analysis [12]. The analyzed surface area (20 × 20 mm^2^) and the fine powdering allowed one to record spectra representative of all the phases which occur in more than ca. 1%.

## 3. Results

### 3.1. Compositions

Table 1 shows the representative compositions measured by SEM-EDS on about a 50 × 50 µm^2^ area. Silica content ranges between 50 and 55 wt %, except for in three spots with lower values (40 and 5 wt %) and one spot with a much higher content (~60 wt %). The low-silica spot corresponds to a grain made of iron oxide (Fe_2_O_3_ = 88 wt %). The alumina content is low (~2–7 wt %), except for in three spots (~13 and 28 wt %). Aluminum arises mainly from clays, since the level of aluminum content in asbestos is low or even nil. The main characteristic is the high content of MgO (~20 wt %), except for in two spots with low content (8 and 12 wt %) and one spot with a very low content (~2 wt %, but also alumina-rich). The CaO content ranges between ~3 and 20 wt %. Small percentage contents of NiO and Cr_2_O_3_ are measured (~0.1 to 0.5 wt %). Obviously, the areas analyzed correspond to different magnesium silicates and to two other phases, namely iron oxide (sample d, spot 6) and calcium aluminosilicate (sample a, spot 8). Vibrational spectroscopic and XRPD analyses will allow the identification of the mineral phases in amounts more than ca. 1%.

### 3.2. Evidence of Asbestos Fibers

**Figure 2:**
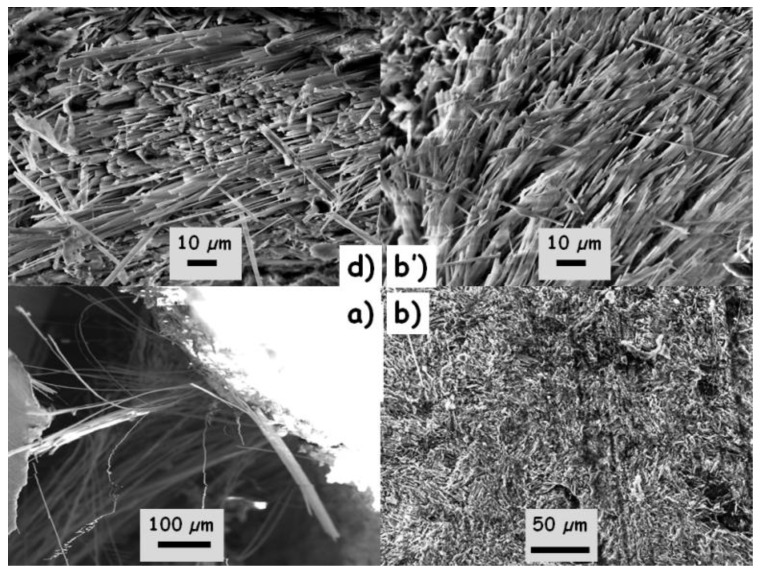
SEM micrographs showing asbestos fiber clusters in broken sections of samples (**a**,**b**,**d**) (see Figure 1); (**a**) large fiber pull-out after fracture; (**b’**) detail of the sliced section of sample b showing the fiber bundles.

Visual and optical microscopic examination can easily detect the presence of long fibers: see in particular, in Figure 1, the right-hand side of sample d (close to the scale) and the top left-hand side of sample b (above the o mark). The beautiful and flat sections obtained by sawing with a diamond saw testify to the good mechanical resistance of the objects, in accordance with the time necessary to saw the shards. This good mechanical strength indicates good sintering. The presence of these fibers is much more obvious on the SEM images (Figure 2). A preferential orientation of the fibers is observed perpendicular to the section of the pottery. This is consistent with a shaping on a potter′s wheel, which orients the bundles of fibers by the turning movement and pressure of the hands. The diameters of the individual fibers range between about 100 nm and 1 µm, and the length can reach a few centimeters. The fibers form heaps and clumps, and big grains are visible (see, e.g., Figure 1a, spots 3, 4, 8 and 6). The red color of the body, especially on the periphery, indicates that firing has been undertaken under an oxidizing atmosphere. The porosity of the body is high, as expected for terracotta fired at a low temperature, i.e., at a temperature inferior to the formation of a large amount of a liquid phase, which would typically be 1050 °C for CaO and MgO aluminosilicates [13].

**Figure 3:**
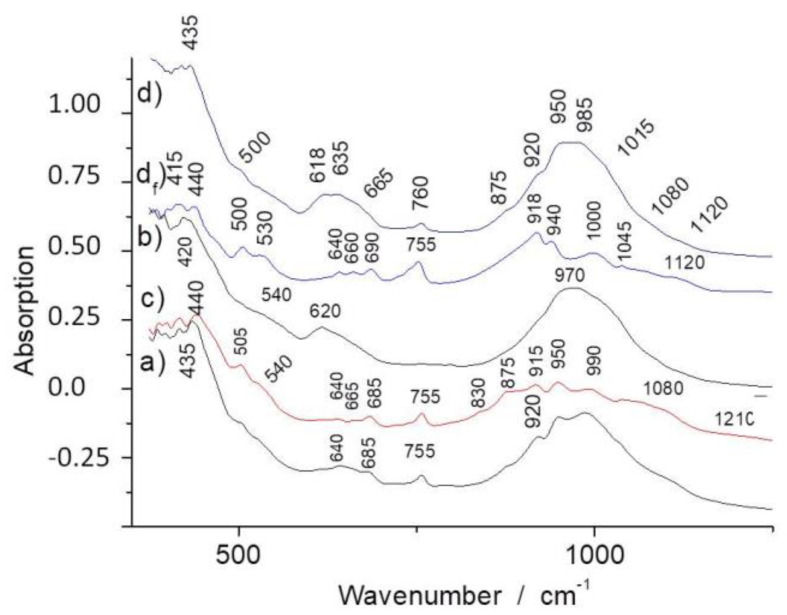
FTIR ATR spectra of samples (**a**–**d**) (see Figure 1) in the silicate framework spectral range; (**d_f_**) spectrum recorded on extracted asbestos fibers.

**Figure 4:**
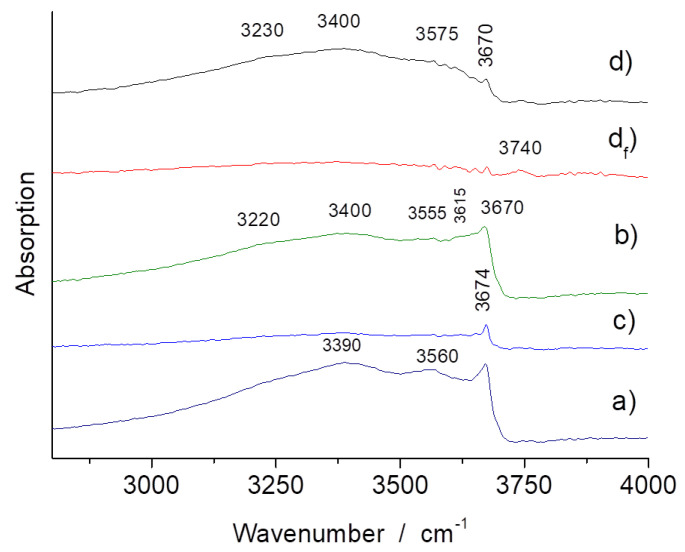
FTIR ATR spectra of samples (**a**–**d**) (see Figure 1) in the O-H/H_2_O spectral range; (**d_f_**) spectrum recorded on extracted asbestos fibers.

Figure 3 and Figure 4 show representative FTIR ATR spectra collected on powdered fragments of the shards. Spectrum d_f_ has been recorded on a fiber bundle extracted from sample d. The bands, for instance, the Si-O stretching and bending modes at ~960 (1015 and 1080) and 620 cm^−1^ (Figure 3), are characteristic of asbestos and similar compounds (amphiboles and pyroxenes) [14,15,16,17,18,19,20,21]. From this first analysis, it can be seen that the samples b and d on the one hand and the samples a and c on the other hand are rather similar, but sample d appears to be more heterogeneous. The narrow O-H stretching modes at ~3670 cm^−1^ (Figure 4) are very specific and fit well with asbestos. On the contrary, the broad O-H band peaking at ~3400 cm^−1^ with a d shoulder at 3200 cm^−1^ is assignable to water traces adsorbed at the pore surface, as observed for many ceramics.

**Figure 5:**
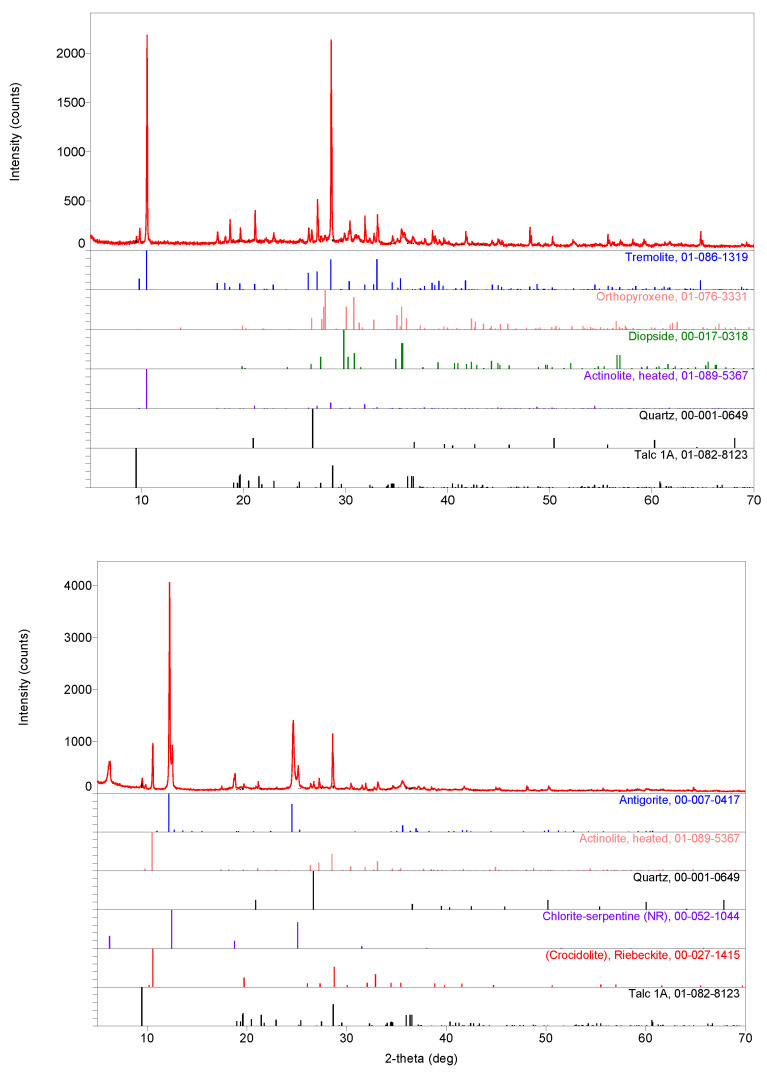
XRPD patterns for samples c (**top**) and d (**bottom**). XRPD patterns of indexed phases: antigorite (PDF# 00–007-0417), heated actinolite (PDF# 01-089-5367), quartz (PDF# 00-001-0649), chlorite–serpentine (NR) (PDF# 00-052-1044), crocidolite (riebeckite) (PDF# 00-027-1415), tremolite (PDF# 01-086-1319), orthopyroxene (PDF# 01-076-3331), diopside (PDF# 00-017-0318) and talc 1A (PDF# 01-082-8123) are presented in the form of straight intensity lines at the pattern bottom [21,22,23,24,25,26,27,28,29]. See Appendix A for XRPD patterns of samples a and b.

Differences between samples are evident; for instance, the spectrum of sample c looks to be richer in asbestos fibers, in accordance with the XRPD results (Figure 5, see further). These differences could arise from the use of different raw materials and/or different relative amounts of fibers and clay used in the preparation and also from different degrees of reaction/transformation under heating.

### 3.3. Tentative Identification of Minerals

There is a large variety of fibrous silicates and silicates built with silicate chains, isolated (inosilicates) or connected to form layers (phyllosilicates). Serpentinite minerals (corresponding rock: serpentine), of an “idealized” formula Mg_3_Si_2_O_5_(OH)_4_, are hydrous phyllosilicates. Many polymorphs are identified; the most frequent minerals are antigorite, chrysotile and lizardite. Five types of amphiboles, belonging to the inosilicate group but with rather similar composition (with orthorhombic or monoclinic unit-cells), are common and often associated with serpentinites: crocidolite (Na_2_(Fe,Mg)_3_Fe_2_Si_8_O_22_(OH)_2_), amosite ((Mg,Fe)_7_Si_8_O_22_(OH)_2_), tremolite (Ca_2_Mg_5_Si_8_O_22_(OH)_2_), actinolite (Ca_2_(Mg,Fe)_5_Si_8_O_22_(OH)_2_) and anthophyllite ((Mg,Fe)_7_Si_8_O_22_(OH)_2_). All these phases contain silicon, calcium and iron as main elements, as can be seen in the compositions given in Table 1. Nephrite (Ca_2_(Mg,Fe)_5_Si_8_O_22_(OH)_2_), a rock rich in amphibole, is used as jade. Pyroxenes, e.g., diopside (CaMg(SiO_3_)_2_), also belong to the inosilicate group but exhibit a simpler composition, without the OH group. Orthopyroxenes have a more complex formula: (Mg,Fe)_2_Si_2_O_6_. Jadeite, the second form of jade, has a composition close to NaAlSi_2_O_6_. Due to the similarity of the XRPD patterns of the serpentinites and amphibole polymorphs [22,23,24,25,26,27,28,29,30], Raman spectroscopy is advantageous for mineral identification [31,32,33,34,35,36,37,38,39,40,41]. The identification of specific minerals remains difficult, especially when their structures are degraded by firing in the kiln.

Inosilicates are built with [SiO_3_]^2−^ entities forming long chains. The amphibole structure is composed of the [Si_4_O_11_(OH)]^7−^ anion (i.e., four of the above entities, but also hydroxylated). The Raman spectrum of these phases, characterized by a short Si-O-Si bridge between adjacent tetrahedra, shows a well-defined stretching mode varying from 650 to 690 cm^−1^ (see further), or even up to 700 cm^−1^ for jadeite in which aluminum replaces part of the silicon framework.

The main forms are serpentine, lizardite and chrysotile (called “white asbestos”), which have been used mostly in modern building and automotive applications [41] due to the high quality of the fibers (in terms of length, small diameter, etc.); the other main form is antigorite. Magnesium ions can be replaced by other elements such as iron, nickel, titanium and chromium. Crocidolite is called “blue asbestos” and has been selected for thermal insulation [42].

**Figure A1:**
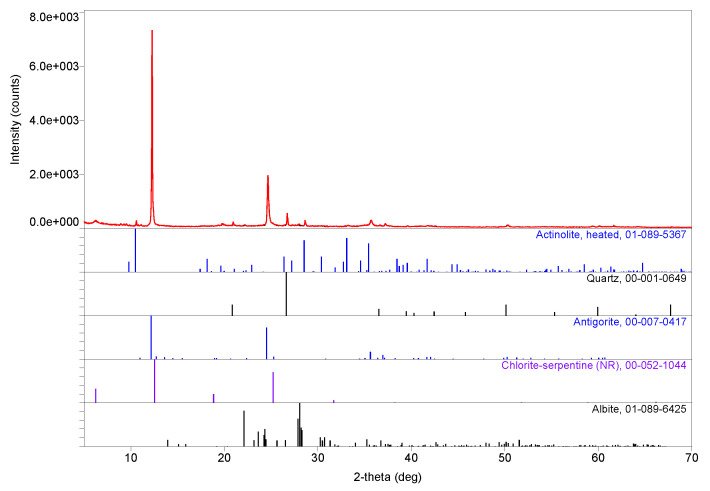
XRPD pattern for specimen a. XRPD patterns of indexed phases: heated actinolite (PDF# 01-089-5367), quartz (PDF# 00-001-0649), antigorite (PDF# 00-007-0417), chlorite–serpentine (NR) (PDF# 00-052-1044) and albite (PDF# 01-089-6425) are presented in the form of straight intensity lines at the bottom of the pattern.

**Figure A2:**
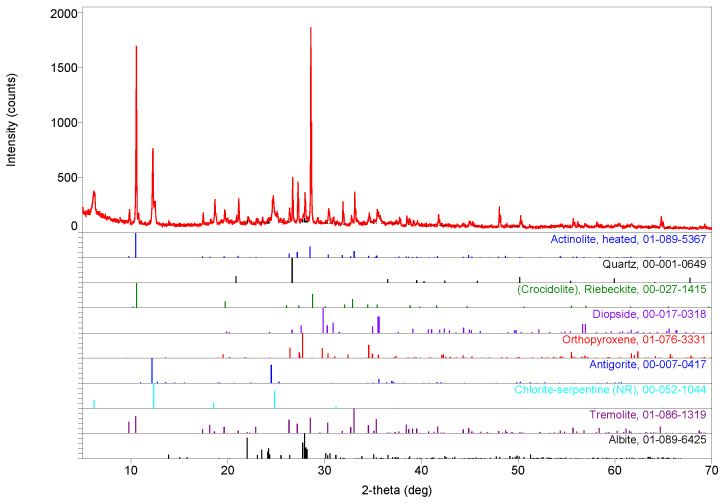
XRPD pattern for specimen b. XRPD patterns of indexed phases: heated actinolite (PDF# 01-089-5367), quartz (PDF# 00-001-0649), crocidolite (riebeckite) (PDF# 00-027-1415), diopside (PDF# 00-017-0318), orthopyroxene (PDF# 01-076-3331), antigorite (PDF# 00-007-0417), chlorite–serpentine (NR) (PDF# 00-052-1044), tremolite (PDF# 01-086-1319) and albite (PDF# 01-089-6425) are presented in form of straight intensity lines at the bottom of the pattern.

With the aid of the PDXL 2 software, it is possible to identify a larger amount of tremolite and minor amounts of diopside, actinolite, talc (all magnesium-based aluminosilicates) and quartz in sample c (Figure 5 top). It is also possible to identify large amounts of antigorite and chlorite–serpentine and minor amounts of (heated) actinolite, crocidolite (riebeckite), talc and quartz in sample d (Figure 5 bottom). Talc (Mg_3_Si_4_O_10_(OH)_2_) results from the degradation of amphiboles and pyroxenes and belongs to the phyllosilicate group, like the serpentinites. XRPD patterns of samples a and b (Appendix A, Figure A1 and Figure A2) are rather similar except for the identification of albite (sodium feldspar, belonging to the tectosilicate group) in both samples in place of orthopyroxene in sample c. Actinolite and quartz are also found in all samples. Antigorite and chlorite–serpentine are found in samples a, b and d, while albite is found only in samples a and b. Crocidolite is found in samples b and d, while tremolite is found in samples b and c. Diopside and orthopyroxene are found in samples b and c, while talc is found in samples c and d. Minor abundant mineral phases noticed in the Raman spectra (see further) were not detected in the XRPD patterns. From the XRPD patterns, their content could be estimated to be less than 3% each.

Two types of raw material sets are recognized, one used for samples a and b and the other used for samples c and d. It should be noted that quantitative analysis of the collected XRPD patterns is not completely reliable due to the preferential orientation of the fibrous phase in relation to the shaping of the pottery.

However, it is obvious that the major component in sample a is antigorite; sample b is primarily composed of actinolite and/or tremolite and/or crocidolite (actinolite, tremolite and crocidolite are difficult to distinguish in complex XRPD patterns like this one), antigorite and chlorite–serpentine; sample c is primarily composed of actinolite and/or tremolite (actinolite and tremolite are difficult to distinguish in complex XRPD patterns like this one); and sample d is primarily composed of actinolite and/or crocidolite (actinolite and crocidolite are hard to distinguish in complex XRPD patterns like this one), antigorite and chlorite–serpentine. Other mineral phases noticed on the XRPD patterns of samples a–d could be treated as moderately abundant.

**Figure 6:**
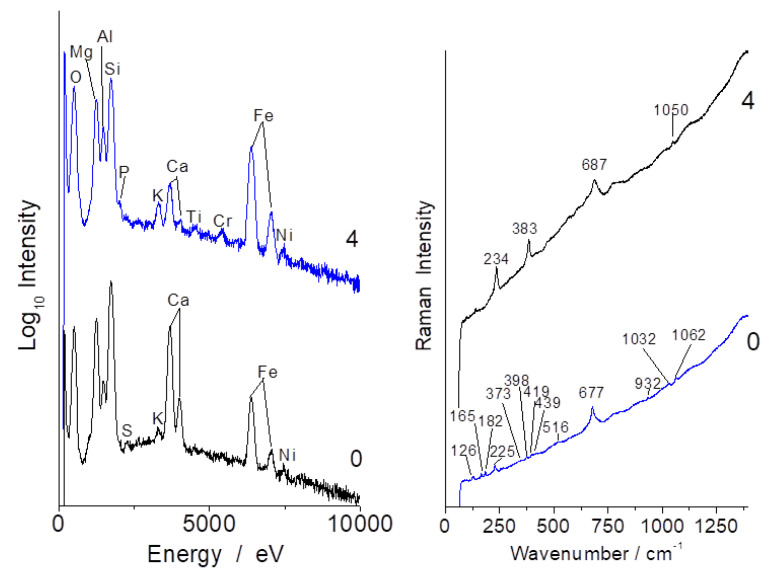
Representative EDS (**left**) and Raman (**right**) spectra recorded on spots 0 and 4 of sample b (see Figure 1).

Figure 6 shows representative Raman spectra recorded from the white fiber yarns located at the top left of sample b (spectrum #0) and on the section of a large grain (#4).

**Figure 7:**
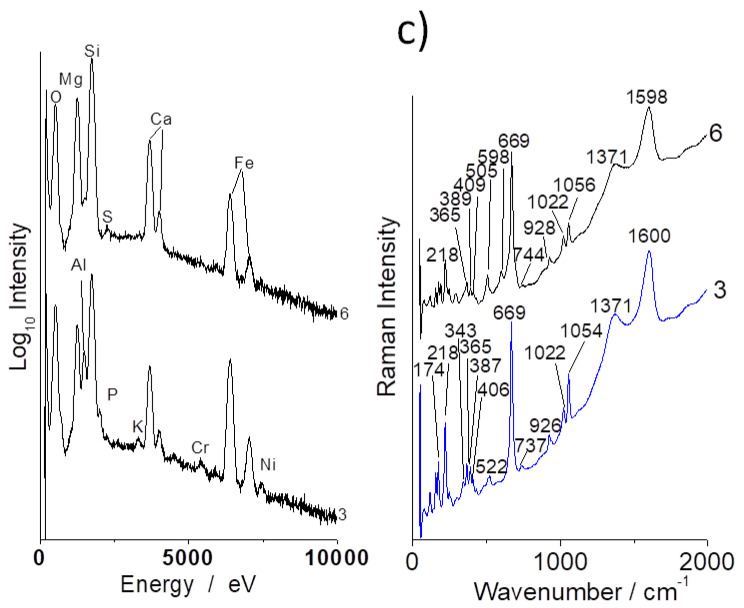
Representative EDS (**left**) and Raman (**right**) spectra recorded on some spots of sample c (see Figure 1).

**Figure 8:**
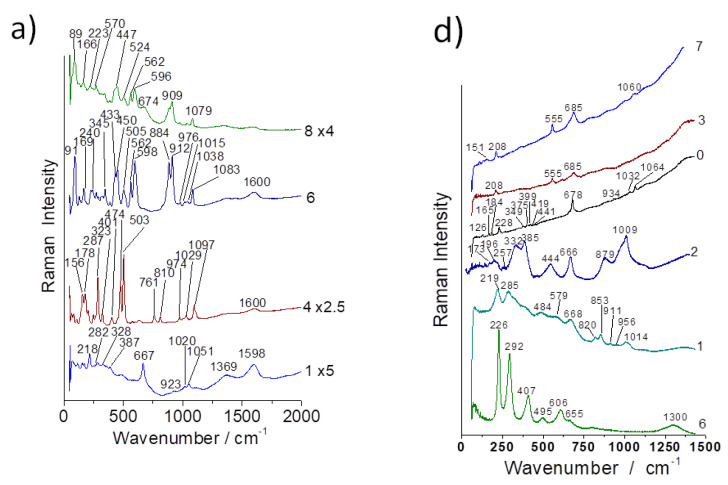
Representative Raman spectra recorded on some spots of samples a (**left**) and d (**right**) (see Figure 1).

**Figure A3:**
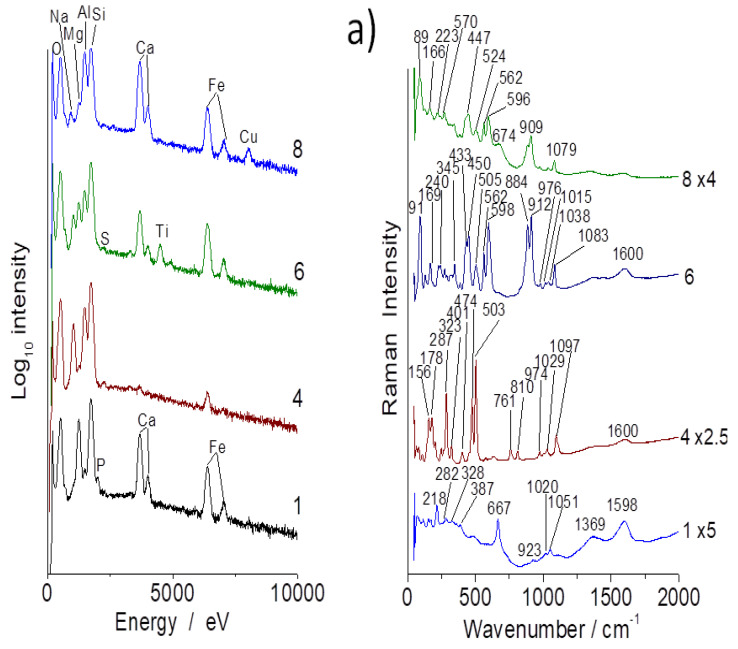
Representative EDS (**left**) and Raman (**right**) spectra recorded on some spots of sample a (see Figure 1).

**Figure A4:**
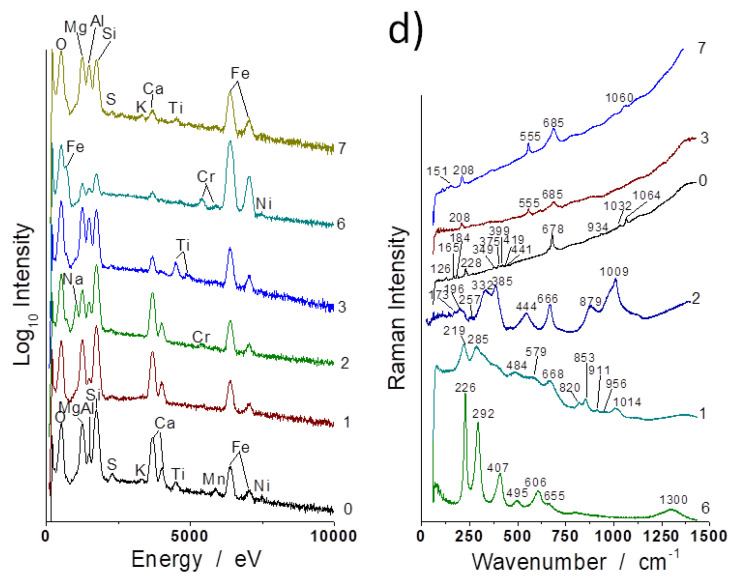
Representative EDS **(left**) and Raman (**right**) spectra recorded on some spots of sample d (see Figure 1).

The spectra show the characteristic signature of serpentinite (ca. 230, 390 and 680 cm^−1^ peaks corresponding to M-O-Si and Si-O-Si vibrations) [33]. Raman signatures collected on the different samples (Figure 6, Figure 7, Figure 8, Figure A3 and Figure A4) look rather similar but band wavenumber shifts of the main band (Si-O-Si stretching mode) and differences in the νO-H patterns (3300–3800 cm^−1^) are obvious.

**Figure 9:**
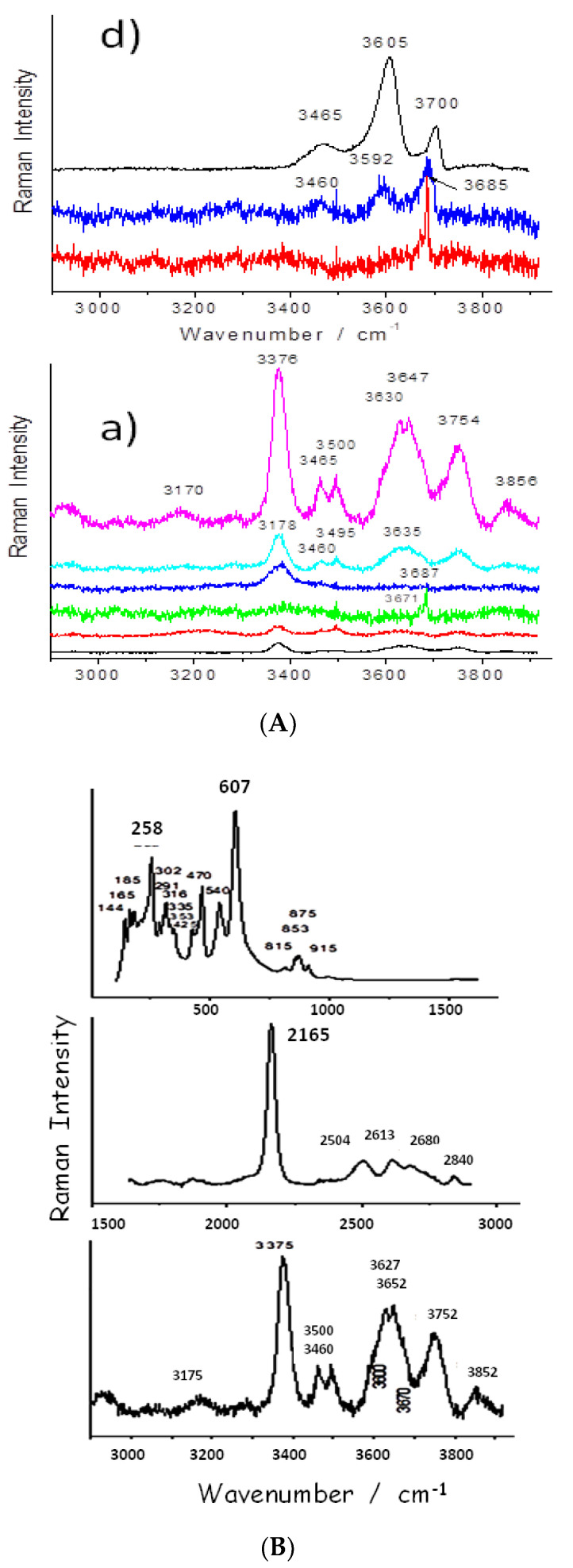
Representative Raman spectra recorded in the 2900–3950 cm^−1^ range on samples a and d (**A**, above, see Figure 1). Examples of untransformed grain core are given on the top spectra. Maximum intensity is measured in the center of the biggest grains visible on the cross-section. Details for the spectrum of sample a are given on the bottom (**B**).

For instance, the Si-O-Si bridge mode shifts from ca. 658 cm^−1^ in amosite (“brown asbestos”, used in cement and pipe insulation [42]) or 664 cm^−1^ in crocidolite (blue asbestos) to 692 cm^−1^ in chrysotile (white asbestos) [22,25,26]. The differentiation from the infrared spectrum is less clear-cut due to the broader bandwidth of the IR modes and because the global analysis superimposes the contribution of other phases (Figure 3). The bandwidth of the main Raman Si-O-Si stretching mode also shows various bandwidths, from very narrow (Figure 7) to rather broad (Figure 6 and Figure 9).

Chrysotile (white asbestos) consists of long, flexible fibers, the best quality from the point of view considering the fiber’s mechanical properties (the smaller the fiber diameter, the higher the radius of curvature) and the tolerance of the fiber to folding; the fiber quality of blue and brown asbestos is generally lower, and anthophyllite and tremolite are not really as fibrous as amphibole asbestos [38]. The identification of blue and white asbestos in these archaeological potteries indicates that a selection of the best quality available has been made by the potter to optimize the mechanical behavior of the cooking wares.

Important differences are observed in the Raman signature recorded in many places of the samples, especially on coarse grains (Figure 6, Figure 7, Figure 8 and Figure 9). The carbon doublet (ca. 1360–1600 cm^−1^) is observed in all samples, especially in samples a (Figure 7) and c (Figure 8). This carbon can arise from different origins: (i) the transformation of organic residues during the use of the utensils, (ii) the use of carbon-rich clays (clays with organic (humic) acids exhibit a high plasticity) and/or (iii) the firing was undertaken under reducing conditions.

The spectrum characteristic of garnet (strong stretching Si-O mode at ca. 900 cm^−1^ [43]) is observed in sample a, spot 6, supporting the rather high level of aluminum found there.

### 3.4. OH Groups, Water and Clays

Modes characteristic of O-H vibrations are only detected in grain cores (Figure 9), which is ascribed the de-hydroxylation induced by the firing: the bulk temperature of a grain is fixed by the internal reaction until the reaction front has reached its core. Therefore, the pristine materials are preserved in the center of the bigger and more thermally stable grains. The relative intensity of the different components and their peak positions are different from those reported in the literature, with some band wavenumbers being rather close to those of antigorite (3650–3750 cm^−1^) and chrysotile (3700 cm^−1^). Both phases are identified on the XRPD spectra (Figure 4, Figure A1 and Figure A2). Table 2 summarizes the phases identified.

The FTIR spectra of asbestos in the O-H and H_2_O stretching range (3000–4000 cm^−1^) only show narrow peaks characteristic of O-H vibrations [43]. In our case, strong and broad ~3200 and 3400 cm^−1^ bands are dominant, which are typical of water adsorbed on porous oxides [44] and of more or less dehydrated clays [45]. As the volume analyzed by Raman scattering is much more precise, we will discuss the proton species by mainly considering the Raman spectra.

Ethnological studies [7] reports that 3 volumes of clays were mixed with 1 volume of asbestos during the manufacture. Both the XRPD and Raman techniques show no evidence of clay minerals. However, the Raman signature of clays is always difficult to record, and the firing must have degraded the clays sufficiently to make the X-ray diffraction not efficient. The presence of a clay-based product, thermally degraded, is only indirectly identified by IR spectroscopy with the broad signature of adsorbed water at high wavenumber (Figure 4) and by the broad Si-O stretching component at about 970 cm^−1^ (Figure 3), with the latter feature not being observed in the fiber bundle spectrum (Figure 3d_f_).

Surprisingly, the signature of quartz (strong narrow Si-O bending peak at ~465 cm^−1^), a very common mineral of a pottery body, is not easily detected by Raman scattering, although it is present as a minor phase in the XRPD (Figure 5, Figure A1 and Figure A2). On the contrary, feldspar (main Si-O bending mode at ~505 cm^−1^) is observed in many places by Raman scattering (see, e.g., Figure 8).The signature of partially substituted hematite (peaks at ~225, 290, 405, 605 and 1300 cm^−1^ [41] in Figure 8) is also frequently identified in the spectra, in agreement with the high content of iron oxide measured (Table 1; for instance, the approximate percentage of Fe_2_O_3_ for spot 6).

### 3.5. Heating-Induced Effects and Remarks on Preparation Procedure

Most of the spectra exhibiting a serpentinite-like signature show a rather broad ca. 680 cm^−1^ peak (full width at half height between 50 and 100 cm^−1^, as in Figure 5 (sp4) and Figure 8 (sp3)), although the FWHH of the pristine fiber is less (close to ~30 cm^−1^ (Figure 7 [32])). The broadening indicates a distribution of Si-O-Si bond lengths that could have arisen from the degradation of the pristine structure upon heating. The large number of phases identified by XRPD and the variety of Raman spectra observed are consistent with a firing taking place at low temperature, with a resultant degradation of the pristine mineral occurring without the clear formation of neophases. Trittschack et al. [18,19,20] observed the intensity decrease of the O-H stretching mode at ~3600–3700 cm^−1^ over 450 °C (de-hydroxylation) and then the formation of forsterite at a temperature over ~500 °C, with the characteristic doublet seen at ~820–850 cm^−1^.

Complete de-hydroxylation requires heating above ~700 °C. Comparison of the IR patterns in Figure 3 shows that the lowest OH content, based on the intensity of the 3670 cm^−1^ narrow band, is measured for the c and d samples, and the lowest water content is also found for these samples (measured by the lower intensity of the 3200 cm^−1^ broad band). The 3400 cm^−1^ component may arise from the clay-based matrix [45] being more or less transformed by the firing. We can expect that the lowest water content is related to a lower porosity and hence to the higher firing temperature, with the mean composition of the shards being rather similar in a first approach. Indeed, the sample d spectrum measured at spot 2 shows a strong and broad ~1010 cm^−1^ band, consistent with a silicate amorphous phase formed in the reaction. Furthermore, for this sample, it was not possible to observe the fiber bundles by optical and scanning electron microscopy, which is most likely due to the higher degree of reaction between phases.

Figure 9 compares the spectra recorded from the periphery to the center of two large grains in samples a and d. Only in the center is a nice spectrum obtained, and the spectra recorded from the center to the periphery become progressively very noisy due to the degradation that becomes more and more important when approaching the surface of the grain. The firing conditions (temperature, heating rate, levels, etc.) therefore limit degradation, and the fibers are generally preserved, as evidenced by the pull-out over lengths on the order of several millimeters. This testifies to an empirical mastery of the manufacture of CMC.

Observation of a black core or side for all samples (Figure 1), as well as the carbon doublet (1370–1600 cm^−1^) in many places, is consistent with a heating process under reducing conditions; reducing conditions promote the formation of a liquid phase at lower temperatures in iron-rich pottery [13].

### 3.6. Evidence of Residues: Gold Ore Processing

Figure 9 shows a strong narrow peak at 2165 cm^−1^, which is characteristic of a CN bond; this can be ascribed to the incorporation of the conservation chemicals (paraloid, glues) commonly used by archaeologists to preserve the samples. However, cyanoacrylate spectra show a characteristic band at a lower wavenumber, ca. 2250 cm^−1^ [46]. The observed wavenumber here fits very well with the species KAu(CN)_2_ (2165 cm^−1^) and is also not far from that of KAg(CN)_2_ (2141 cm^−1^) [47,48,49]. KAu(CN)_2_ and KAg(CN)_2_ are so-called cyanidation compounds which have been used in gold- and silver-plating/extraction since the 19th century [50]. This would indicate that asbestos-based pottery pieces have also been used as crucibles due to their good resistance to thermal shock. Gold mining is reported to occur locally and not far away from the asbestos outcrops [51]. The demonstration of the possibility of identifying the residues of metallurgical use in archaeological shards opens a new field of research, as this type of analysis has only been conducted to assess potential domestic uses (food residues).

## 4. Conclusions

Two types of raw materials have been identified in these archaeological asbestos-fiber-reinforced ceramic matrix composites, confirming the ethnological classification. It appears that a selection of the most appropriate asbestos fibers has been made by ancient potters. Obviously, the good mechanical strength due to the fiber reinforcement (the fibrous behavior of the length of the fiber pull-out reaches the millimeter range) was selectively searched for, and at that time the toxicity of asbestos fibers was not recognized. This demonstrates that the use of natural eco-friendly products (clays and stones) and their traditional preparation by women (in the ethnological record) are not guarantees of the achievement of a good product. Detailed analysis by Raman microscopy of the νO-H modes shows that the firing conditions were close to or slightly exceeded the degradation temperature of the asbestos fibers, thus preserving their mechanical properties.

The observation of traces of KAu(CN)_2_ proved the use of one pottery shard as a crucible for gold extraction/separation. This highlights the potential of Raman microscopy, a noninvasive and mobile technique, in identifying residues testifying to nondomestic, chemical and metallurgical uses in the case of these ceramics. A sorting of useful shards for additional analyses can therefore be done on site, in museum reserves or on excavation sites.

## Figures and Tables

**Table 1:** Oxide composition measured by SEM-EDS on spots indicated in Figure 1 for samples a to c. Characteristic values in bold.

Oxide	Sample b		Sample c		Sample a			Sample d			
	«0»	«4»	«3»	«6»	«1»	«6»	«8»	«0»	«1»	«2»	«6»
SiO_2_	50.73	**42.79**	50.41	56.78	54.91	55.68	**42.21**	55.52	55.39	**61.24**	**4.68**
Al_2_O_3_	3.47	**11.01**	7.63	1.88	1.63	**14.13**	**28.29**	3.19	1.92	4.88	1.26
CaO	**17.87**	2.43	6.44	9.74	**12.81**	6.34	**20.08**	**11.47**	**13.06**	**11.57**	0.82
MgO	19.74	23.23	15.84	23.53	21.99	**8.02**	**1.93**	22.54	20.03	**11.72**	3.45
K_2_O	0.35	0.97	0.29	0.15	0.18	0.23	0.09	0.20	0.13	0.05	0.09
Na_2_O	0.36	0.31	0.12	1.10	0.76	**4.82**	0.24	0.46	0.76	**5.11**	0.03
Fe_2_O_3_	6.44	**17.75**	**18.12**	6.14	7.18	8.78	6.45	4.54	5.03	4.81	**88.20**
TiO_2_	0.13	0.40	0.18	0.07	0.10	1.52	0.15	0.63	0.08	0.04	0.15
NiO	0.41	0.52	0.44	0.14	0.09	0.04	0.09	0.27	0.12	0.06	0.30
MnO_2_	0.14	0.09	0.17	0.15	0.18	0.24	0.16	0.37	0.08	0.09	0.19
SO_3_	0.14	0.05	0.03	0.22	0.05	0.16	0.18	0.70	0.26	0.20	0.06
Cr_2_O_3_	0.23	0.48	0.34	0.11	0.13	0.05	0.14	0.12	0.15	0.24	0.79

**Table 2:** (**a**) Phases identified by XRPD (estimation of the relative amounts: +, low; ++++, high). (**b**) Phases identified by Raman spectroscopy. Characteristic peaks are indicated (cm^−1^); see Figure 5, Figure 6, Figure 7 and Figure 8 and Figure A1, Figure A2, Figure A3 and Figure A4 in the Appendix A).

(**a**)										
**Samples/ Phases**	**Actinolite (Heated)**	**Antigorite**	**Crocidolite (Blue Asbestos)**	**Tremolite**	**Chlorite–Serpentine**	**Quartz**	**Albite**	**Diopside**	**Ortho-pyroxene**	**Talc**
a	++	++++			++	++	+			
c	++			++++		+		+	++	+
d	+++	++++	++		++	+				+
b	++++	+++	++	+	++	++	+	+	+	
(**b**)										
**Samples/ Phases**	**Antigorite Chrysotile Serpentine**	**Crocidolite Amphibole (Blue Asbestos)**	**Amosite Amphibole**	**Quartz**	**Feldspar**	**Diopside**	**Pyroxene**	**Talc**	**Forsterite**	
	690	~664	658	460	503	668 1015	680 1007		820–850	
a		++		+	+	+				
b	+++	+++								
c		+++			+	+				
d	+++	++					+		++	

Other phases: hematite (d); carbon (a,c).
